# Residual effects of combined vibratory and plantar stimulation while seated influences plantar pressure and spatiotemporal gait measures in individuals with Parkinson’s disease exhibiting freezing of gait

**DOI:** 10.3389/fnagi.2023.1280324

**Published:** 2024-01-09

**Authors:** Warongporn Phuenpathom, Pattamon Panyakaew, Peerapon Vateekul, Decho Surangsrirat, Roongroj Bhidayasiri

**Affiliations:** ^1^Chulalongkorn Centre of Excellence for Parkinson’s Disease and Related Disorders, Faculty of Medicine, Chulalongkorn University and King Chulalongkorn Memorial Hospital, Thai Red Cross Society, Bangkok, Thailand; ^2^Department of Computer Engineering, Faculty of Engineering, Chulalongkorn University, Bangkok, Thailand; ^3^Assistive Technology and Medical Devices Research Center, National Science and Technology Development Agency, Pathum Thani, Thailand; ^4^The Academy of Science, The Royal Society of Thailand, Bangkok, Thailand

**Keywords:** freezing of gait, Parkinson’s disease, Parkinson shoe, vibration, plantar pressure

## Abstract

**Introduction:**

Combined plantar pressure and vibratory stimulation has been shown to decrease freezing of gait (FOG) episodes and improve spatiotemporal gait parameters compared to single stimulation in Parkinson’s disease (PD) patients with FOG. However, the effect of combined plantar stimulations on plantar pressure analysis has never been explored.

**Methods:**

Forty PD patients with frequent FOG were allocated to either FOG shoes embedded with a 100 Hz vibratory stimulation at the Achilles tendons and a soft thickened silicone pad at the hallux and sole, or sham shoes with a non-working vibratory motor and a flat non-pressure silicone pad (20 patients per arm) while seated for 96 s. The objective gait and plantar pressure analysis were measured immediately after the stimulation. Outcomes included the normalized percentage of changes in percent FOG (%FOG) and plantar pressure in the heel-strike and push-off phase that were compared between pre- and post-stimulations.

**Results:**

The FOG shoes group showed significantly decreased %FOG (81.5 ± 28.9% vs. 6.8 ± 22.1%, *p* < 0.001), plantar pressure in the heel-strike (47.8 ± 43.7% vs. 4.3 ± 9.8%, *p* < 0.001), plantar pressure in the push-off (57.7 ± 59.6% vs. 6.2 ± 11.6%, *p* < 0.001), force time integral (FTI) (40.9 ± 32.5% vs. 6.6 ± 17.3%, *p* < 0.001), and decreased heel contact time (19.3 ± 12.3% vs. 22.7 ± 32.5%, *p* < 0.001) when compared to the sham group. There was a strong negative correlation between %FOG and peak plantar pressure (*r* = −0.440, *p* = 0.005), plantar pressure in the heel-strike (*r* = −0.847, *p* < 0.001).

**Conclusion:**

Our study demonstrated that the FOG shoe could decrease FOG episodes by improving the heel-strike pressure, toe push-off and normalized heel-to-toe plantar pressure, suggesting that modification inputs from the peripheral sensory systems might significant improvement in FOG in PD.

## 1 Introduction

Freezing of gait (FOG) is commonly observed in 50.6% of patients with Parkinson’s disease (PD) and its prevalence increases up to 64.6% in the advanced stage (Zhang et al., 2021). It manifests as a brief, episodic inability to generate effective steps despite the intention to walk (Nutt et al., 2011), leading to falls, immobility, and poor quality of life (Bloem et al., 2004; Fasano et al., 2017). The complex pathophysiology of FOG in PD encompasses dysfunction of basal ganglia, brainstem locomotor center, as well as abnormalities in executive function and central sensorimotor integration (Nutt et al., 2011; Takakusaki, 2013; Gao et al., 2020). However, emerging insights suggest that peripheral sensory deficit may play an important role in contribution to FOG due to decrease sensory inputs and abnormal sensory gating to the central nervous systems (Tan et al., 2011; Cowie et al., 2012; Martens and Almeida, 2012; Ehgoetz Martens et al., 2013, 2014; Marquez et al., 2020). Thus, diverse modalities of peripheral sensory stimulations have been developed to improve FOG, including synchronized vibration insoles and automated mechanical peripheral stimulation (Winfree et al., 2013; Pinto et al., 2018; Pollet et al., 2023).

Interestingly, combined vibratory and plantar pressure stimulation, involving vibratory stimulation at the bilateral Achilles tendon and pressure stimulation at the left and right first hallux, first metatarsal joint, and sole, has shown promise in significantly reducing FOG severity, and improving stride length in pre-FOG and FOG period, stride velocity, and gait asymmetry in PD patients with FOG compared to single vibratory or pressure stimulation (Phuenpathom et al., 2022). The proposed mechanisms included the synergistic effects of different peripheral sensory inputs to augment the sensory feedback and feedforward loops (Pereira et al., 2016) and the improvement of sensory gating prior to the FOG episodes. However, the effects of combined peripheral sensory stimulations on plantar pressure, contributing to amelioration of FOG episodes have never been explored. Thus, we have developed a therapeutic Freezing of Gait (FOG) shoe with integrated vibratory and pressure plantar stimulation that will emphasize the impact of the combined peripheral plantar stimulation integrated in the FOG shoe on plantar pressure as well as FOG episodes and spatiotemporal gait parameters. This might lead to a better understanding of the crucial involvement of the peripheral nervous system, particularly the changes in plantar pressure, in the pathophysiology of FOG.

Assessing plantar pressure distribution through gait analysis provides insights into peak pressure dynamics throughout the gait cycle (Orlin and McPoil, 2000). Patients with PD demonstrated reduced peak forces at the forefoot and heel, along with increased midfoot load compared to healthy controls, indicating a decreased heel-strike (Nieuwboer et al., 1999). This may be attributed to impaired anticipatory postural adjustment (APA) prior to the initiation of gait. In addition, slower pressure load on the heel was followed by a flat roll-off and the early forefoot loading. These sequential changes contribute to delayed push-off and worsening of gait velocity in PD relative to the control group, giving rise to the distinctive festination and shuffling gait patterns observed in PD patients (Nieuwboer et al., 1999). Furthermore, a study involving PD patients with histories of FOG revealed reduced peak pressure at the hallux area. This outcome is attributed to increased activation of tibialis anterior and toe extensor muscles, resulting in inadequate push-off and compromised heel-strike initiation, consequently triggering FOG (Zou et al., 2023). However, research on plantar pressure dynamics in PD patients exhibiting FOG during walking assessments and after plantar stimulation is limited. Exploring the effects of combined vibratory and pressure stimulation on plantar pressure during heel-strike and push-off offers insights into how FOG shoes may alleviate FOG at the peripheral level.

In this study, we aim to explore the immediate effect of the FOG shoes integrated with combined vibratory and pressure plantar stimulations while seated on FOG episodes, plantar pressure, and spatiotemporal gait parameters during or prior to FOG in PD patients with FOG. Furthermore, we would like to investigate the impact of FOG shoe on the underlying peripheral mechanisms driving FOG and the effect of combined peripheral plantar stimulation on plantar pressure, particularly during the heel-strike and toe-off phases. We hypothesized that applying FOG shoes integrated with combined static vibratory and pressure stimulation while seated would increase the heel-strike pressure and normalize the forefoot/heel pressure as well as improve spatiotemporal gait parameters that lead to immediately reduce FOG and our FOG shoe might be a new option for the treatment of FOG in PD.

## 2 Materials and methods

### 2.1 Participants

Forty individuals diagnosed with PD based on the criteria established by the Movement Disorder Society (MDS) were identified from the Outpatient Clinic of the Chulalongkorn Centre of Excellence on Parkinson’s Disease and Related Disorders (ChulaPD, www.chulapd.org) (Postuma et al., 2015) between December 2021 to May 2022. The criteria for inclusion consisted of individuals with PD who experienced frequent FOG, validated by a score exceeding 1 on Item #3 of the FOG-Questionnaire (FOG-Q) (“Do you feel that your feet get glued to the floor while walking, making a turn or when trying to initiate walking?”) (Giladi et al., 2000). Prior to the study, individuals were required to adhere to an unchanged antiparkinsonian medication schedule for a minimum of three months and achieve a score higher than 23 on the Thai Mental State Examination (TMSE). Exclusion criteria included Hoehn and Yahr (H & Y) stage 5, significant peripheral neuropathy, significant musculoskeletal or cardiovascular diseases, respiratory disorders, uncorrected visual impairments, and use of specific medications affecting walking ability (i.e., benzodiazepine or anti-cholinergic drugs). Approval for the research was granted by the Human Ethics Committee at Chulalongkorn University’s Faculty of Medicine (Institutional Review Board [IRB] No. 097/64). Written informed consent was obtained from all participants. The patients who participated this study were not similar to whom enrolled in our previous study (Phuenpathom et al., 2022).

### 2.2 Experimental protocol

Participants were randomly assigned to either the FOG shoe group or the sham shoe group in a 1:1 ratio. Blinded allocation and assignment of interventions were conducted. Stimulation was administered during the “on” phase after administration of dopaminergic agents. (Confirmed by the symptoms of patients within 2 h after administration of dopaminergic agents).

**Figure 1:**
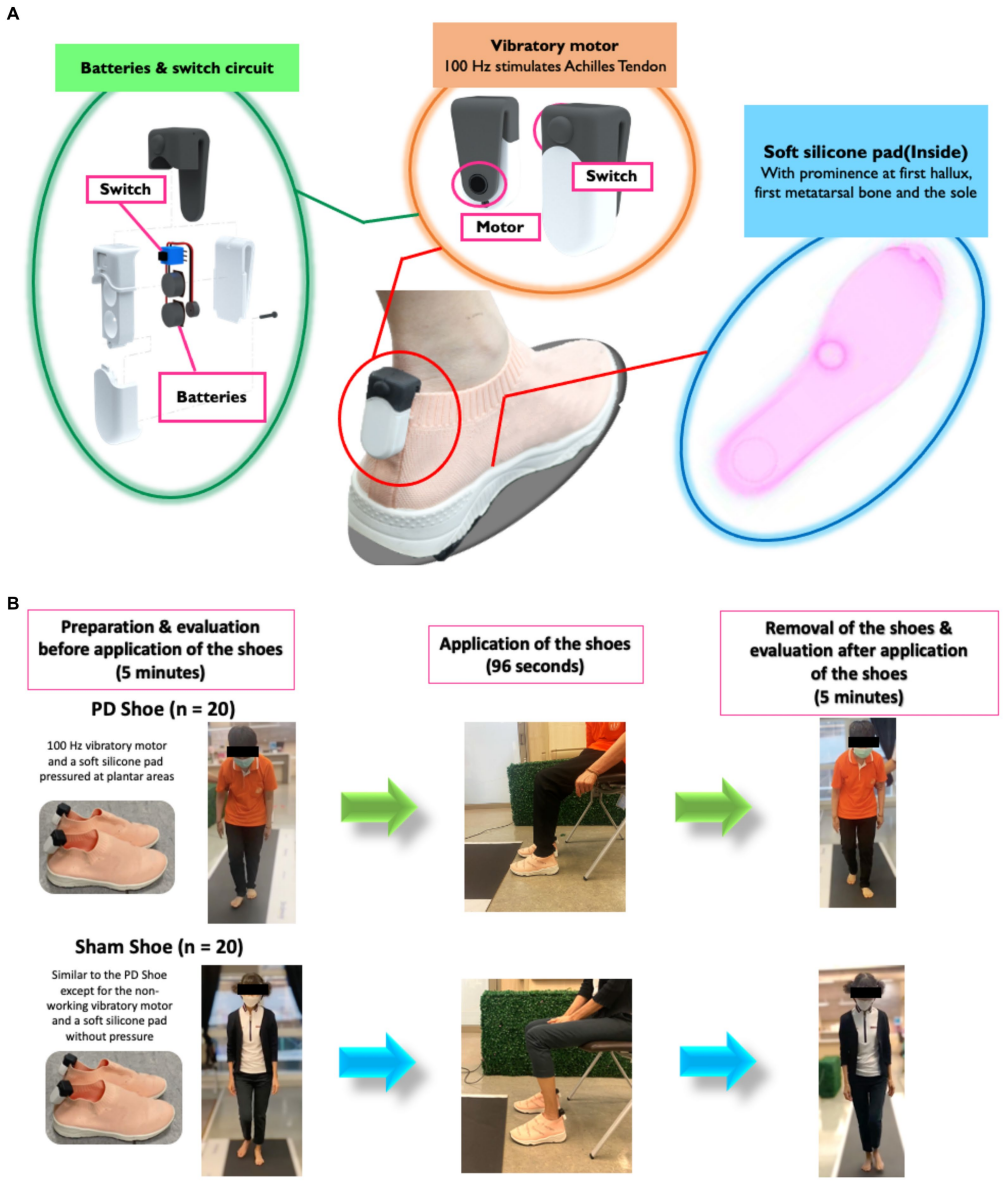
**(A)** The FOG shoe and its components. **(B)** Treatment procedure.

Vibratory and pressure stimulations were simultaneously delivered using prototype FOG shoes comprising a 100-Hz vibratory motor placed bilaterally at the Achilles tendon (De Nunzio et al., 2010; Phuenpathom et al., 2022); 3 V batteries with a switch circuit connected to a vibrator motor; and a soft silicone pad with thickened silicone at the first hallux (1×1.2×1 cm^3^), the first metatarsal bone (2 × 3 × 2 cm^3^), and the sole (0.5 × 3.5 × 3.5 cm^3^). The upsole of the shoe was made of polyester, while the midsole and outsole were rubber (Figure 1A). This protocol was established based on our previous study (Phuenpathom et al., 2022). In brief, the 100-Hz vibratory stimulation for 96 s was designed to stimulate muscle spindles, causing an illusory stretching sensation and contraction of antagonistic muscles to move the body forward and increase the sensory feedback to the spinal cord to ameliorate FOG (De Nunzio et al., 2010; Pereira et al., 2016; Phuenpathom et al., 2022). Simultaneously, the plantar pressure protocol was applied on those selected areas known for their highest-pressure sensitivities, thereby increasing peripheral sensory feedback (Novak and Novak, 2006; De Nunzio et al., 2010; Pereira et al., 2016). The sham shoes were made of similar materials and shared a comparable shape with the FOG shoes. However, they featured a non-functioning vibratory motor (batteries removed) and internal flat silicone pads (Figure 1B). The size of the experiment shoes was matched with individual shoe size in all participants. For the FOG shoe group, a 96-s stimulation was administered to seated patients, while the sham shoe group underwent an identical process as the FOG shoe group, except for the stimulation (Kleiner et al., 2018; Phuenpathom et al., 2022). The evaluation of spatiotemporal gait parameters and plantar pressure were performed on the Strideway^®^ System (Version 7.8, Tekscan, Inc., Boston, MA, United States) both at the baseline and within 5 min after the completion of stimulations (Melai et al., 2011). All participants were instructed to walk at their normal pace three times on the gait mat for 5.25 meters each time. All steps taken during the three trials were collected and averaged (Figure 1B). The peak plantar pressure was calculated in all walking steps including the FOG.

## 3 Study outcomes

### 3.1 Clinical demographics, responses to questionnaires, and rating scales

The clinical profiles of all enrolled participants consisted of essential factors, including age, gender, height, shoe size, H & Y stage, disease duration, TMSE score, and levodopa equivalent daily dose (LEDD). These characteristics were recorded and subjected to comparative analysis across the study cohorts. The assessment of FOG-Q was performed by all participants to corroborate their consistent experience of freezing episodes (Gilat, 2019). Additionally, with permission from the Movement Disorder Society (MDS), the Thai-translated adaptations of the MDS-Unified Parkinson’s Disease Rating Scale (MDS-UPDRS) part III during the “on” period and the Timed Up and Go (TUG) test were employed to establish baseline measurements against those obtained immediately post-stimulation (Jagota et al., 2022).

### 3.2 Objective outcomes

The primary outcome of this study centered on the assessment of FOG severity and quantified through the calculation of the percentage of time spent in FOG (%FOG). This metric was defined as the ratio of the total duration of freezing episodes to the overall gait duration, expressed as a percentage. The beginning of a freezing episode is an unintentional and/or ineffective stepping event lasting more than 1 s (Schaafsma et al., 2003). The end of a freezing episode was established by observing the point at which individuals with FOG executed a minimum of two consecutive effective steps characterized by typical stride length and uninterrupted forward motion (Gilat, 2019). The total duration of freezing episodes was objectively evaluated from the footprint results and confirmed from videos extracted from the Strideway^®^ System (Version 7.8, Tekscan, Inc., Boston, MA, United States). FOG events were detected during walking straight on the gait mat. The secondary outcome was the peak plantar pressure (PPP), which represents foot loading on the ground (Ferber et al., 2013). Other plantar pressure parameters were also recorded as follows: (1) plantar pressure during heel-strike and push-off, which express the maximal load in a specific area under a heel and a forefoot (Nieuwboer et al., 1999; Gilat, 2019; Nonnekes et al., 2019); (2) heel contact time, which reflects the duration of the heel-strike phase (Leal-Junior and Frizera-Neto, 2022); and (3) force time integral (FTI), which demonstrates the cumulative foot loading over time (Buldt et al., 2018). Spatiotemporal gait parameters and plantar pressure were assessed both at baseline and immediately following stimulation, including stride velocity (cm/s), stride length (cm), their respective coefficients of variation (CV), and cadence (steps/min). All outcomes including plantar pressure and spatiotemporal gait parameters were automatically calculated from the Strideway^®^ System (Version 7.8, Tekscan, Inc., Boston, MA, United States). Moreover, attention was directed toward the mean length of the three strides taken immediately before an onset of a freeze and their corresponding CV values. These particular parameters hold significance in characterizing the critical phase preceding a freezing episode, also known as the pre-FOG period (Nieuwboer et al., 1999). All participants were instructed to walk at their normal pace three times on the gait mat for 5.25 m each time (total 15.75 m). All steps taken during the three trials were collected and averaged, including both the acceleration and deceleration components.

## 4 Statistical analysis

The determination of an appropriate sample size was performed by the probabilities of achieving reductions in %FOG within the control and combined peripheral plantar stimulation groups. Specifically, we aimed for reductions of 9 and 63% with a two-sided significance level of 0.05 and a statistical power of 80% (Bernard, 2000; Phuenpathom et al., 2022). Considering a projected dropout rate of 10%, our calculations indicated that a cohort of 35 individuals with PD would be required. Thus, a total of 40 participants were included, equally distributed with 20 in each arm of the study. Baseline characteristics, plantar pressure data, and spatiotemporal gait parameters were summarized using appropriate statistical measures, including means and standard deviations (SDs), or frequencies and percentages, as applicable. An assessment of the normality of distributions for demographic, plantar pressure, and spatiotemporal gait parameter data within each study group was conducted using the Shapiro–Wilk test. To facilitate meaningful comparisons across groups, we calculated and normalized the percentage change in each parameter before and after the application of FOG shoes. This normalization process, achieved through the formula [(Parameters acquired after application of the shoes – Parameters acquired before application of the shoes)/Parameters acquired before application of the shoes] × 100, ensured a consistent and standardized basis for comparison among all outcomes.

For the comparison of demographic and continuous data among groups, Unpaired t-tests or Mann–Whitney *U* tests were used, considering the normality of distribution. For the comparison of categorical data among groups, Chi-square test or Fisher’s exact test were applied. Since the MDS-UPDRS III score and some of pre-stimulation gait and plantar pressure parameters were significantly different between the active and the sham stimulation groups (Supplementary Table 1), the multivariable linear regression analysis with using the pre-intervention parameters including the MDS-UPDRS III scores and objective gait and plantar pressure parameters as covariate was performed to compare the normalized percent changes between groups and adjust the results in a post-hoc analysis. Changes in each spatiotemporal gait and plantar pressure parameters were plotted at the pre and post stimulation to explore the individual response in both the sham and FOG shoe group (Supplementary Figure 1). Exploratory correlations were also conducted using Pearson’s correlation, focusing on objective FOG parameters, plantar pressure analysis, and spatiotemporal gait parameters. Visual representations of the correlations were generated through a correlation heatmap. All statistical analyses were conducted utilizing SPSS software version 23 and R software version 4.2.2. value of ps were calculated to determine the significance of normalized percent change values, with statistical significance defined at *p* < 0.05 in a two-tailed test. The power of the analysis was assessed, and Cohen’s d values were computed to assess the magnitude of effect in cases of significant differences in value of ps. Established thresholds of 0.2, 0.5, and 0.8 were adhered to denote small, moderate, and large effect sizes, respectively.

## 5 Results

Overall, the patients were diagnosed with moderate stages of PD with axial involvement with a mean H & Y stage 2.98 ± 0.60 and a mean disease duration 12.05 ± 3.24 years. In the pre-stimulation phase, all recruited PD patients regularly experienced FOG, defined by the mean FOG-Q scores of 21.15 ± 2.29. There were no significant differences among the groups with respect to demographics, height, shoe size, disease duration, H & Y stage, LEDD, or TMSE scores before stimulation (Table 1). The percentage of change in MDS-UPDRS part III and TUG scores in the FOG shoe group was significantly reduced when compared with the sham shoe group (6.1 ± 4% vs. 3.7 ± 2.9%, *p* = 0.001, Cohen’s *d* = 0.69; 16.8 ± 8% vs. 5 ± 8.3%, *p* < 0.001, Cohen’s *d* = 1.45). The primary outcome that included the normalized %FOG was significantly decreased for the FOG shoe group compared to the sham shoe group (81.5 ± 28.9% vs. 6.8 ± 22.1%, *p* < 0.001, Cohen’s *d* = 2.9) (Table 2).

**Table 1:** Baseline characteristics before PD patients with FOG applied the shoes.

Items	Sham group (*n* = 20)	FOG shoe group (*n* = 20)	*p*-value
Age (years)	71.2 ± 7.2	72 ± 7.2	0.799
Male gender *N* (%)	10 (50.0)	16 (80.0)	0.097
Height (m)	1.65 ± 0.04	1.66 ± 0.04	0.659
United States shoe size	8.4 ± 1.1	8.4 ± 1.3	0.841
Disease duration (years)	12.4 ± 3.2	12.1 ± 3.2	0.659
Levodopa equivalent daily dose (mg)	991.2 ± 245.9	1,079.4 **±** 433.9	0.565
Hoehn and Yahr stage in the “On” period	3 ± 0.6	3.1 ± 0.6	0.341
TMSE scores, mean ± SD	24.6 ± 0.6	24.8 ± 1.1	0.925
FOG-Q	20.7 ± 1.9	21.6 ± 2.6	0.253

Values shown are means ± standard deviations, except for gender. Value of *p*s from Unpaired *t*-test or Mann–Whitney *U* test for continuous data, and value of of *p*s from Chi-square test or Fisher’s exact test for categorical data. *p* < 0.05 considered as statistically significant. PD, Parkinson’s disease; TMSE, Thai Mental State Examination; FOG, freezing of gait; FOG-Q, Freezing of Gait-Questionnaire; SD, Standard Deviation.

**Table 2:** Rating scales, and objective gait parameters before and after PD patients with FOG applied the shoes.

Outcomes	Sham shoe (*n* = 20)			FOG shoe (*n* = 20)			*p*-value^*^	Cohen’s *d*
	Before (Mean ± SD)	After (Mean ± SD)	Normalized percent changes (%)	Before (Mean ± SD)	After (Mean ± SD)	Normalized percent changes (%)		
*Rating scales*								
MDS-UPDRS part III in the “On” period	27.8 ± 2.3	26.8 ± 2.6	3.7 ± 2.9^a^	32.8 ± 9.2	31 ± 9.3	6.1± 4.0 ^a^	0.001	0.69
Time-Up-and-Go (seconds)	17.2 ±1.9	16.4 ± 2.8	5 ± 8.3 ^a^	18 ± 2.2	15.1 ± 2.9	16.8 ± 8 ^a^	<0.001	1.45
*Objective gait parameters*								
Percent FOG (%)	23.9 ± 8.2	24.6 ± 6.6	6.8 ± 22.1^b^	33.6 ±17.7	6.2 ± 9.1	81.5 ± 28.9 ^b^	< 0.001	2.9
Peak plantar pressure (Kpa)	219 ± 9.6	218.2 ± 66.2	0.4 ±29.5^b^	149.7 ± 24	188.3 ± 99.7	27.2 ± 69.1^b^	0.870	–
Plantar pressure in the heel-strike phase (%BW)	28.7 ± 11.5	27.8 ± 13.3	4.9 ± 10.3^a^	32.5 ± 23.6	44.5 ± 33.3	47.8 ± 43.7^b^	< 0.001	1.35
Plantar pressure in the push-off (%BW)	29.6 ± 11	31.4 ± 11.9	6.2 ± 11.6^b^	27 ± 23.7	34.7 ± 20.0	56.3 ± 60.9^b^	<0.001	1.14
FTI (%BW^*^ Seconds)	24.1 ± 9.4	22 ± 9	6 ± 40.9^a^	14.9 ± 13.2	22.7 ± 23.3	40.9 ± 32.5^b^	< 0.001	0.94
Heel contact time (seconds)	0.4 ± 0.2	0.5 ± 0.3	22.7 ± 32.5^b^	0.4 ± 0.1	0.4 ± 0.1	19.3 ±12.3^a^	< 0.001	0.14
Stride velocity (cm/s)	67.9 ± 15.5	68.2 ± 15.8	0.5 ± 2.7^b^	44.8 ± 23.3	66.5 ± 31.1	61.4 ± 55.3^b^	0.003	1.56
Stride length (cm)	70.6 ± 20.4	74 ± 16.4	13.1 ± 41.7^b^	54.8 ± 27.8	71.6 ± 32.9	34.2 ± 31.5^b^	0.315	–
Cadence (steps/min)	96.3 ± 17.9	100.8 ± 14.6	5.6 ± 10^b^	77.5 ±12.1	106 ± 22.2	37 ± 18.1^b^	< 0.001	2.15
Mean stride lengths of three strides before a freeze (cm)	48.3 ± 20.4	69.2 ± 21.1	0.7 ± 9.1^b^	37.9 ±11.4	52.3 ± 22.4	62.7 ± 91.2^b^	0.001	0.96
Coefficient of Variation (CV) of stride velocity (%)	33.4 ± 4.7	33.7 ± 5.4	0.8 ± 6.5^b^	42.8 ±17.7	39.9 ± 10	1.3 ± 24.7^b^	0.01	–
Coefficient of Variation (CV) of stride Length (%)	9.3 ± 8.1	11 ± 9.9	23.3 ± 47.9^b^	18.5 ± 18.9	14.5 ± 13.3	10.6 ± 51.4^a^	0.095	–
Coefficient of Variation (CV) of three strides before a freeze (%)	4.8 ± 1.9	6.1 ± 3.2	53.8 ± 107.7^b^	21.3 ± 25.2	14.9 ± 13.6	5.8 ± 71.8^b^	0.45	–

Normalized percent changes were calculated as [Parameters acquired after application of the shoes – Parameters acquired before application of the shoes/Parameters acquired before application of the shoes] × 100.

a percent decrease.

b percent increase.

* value of ps from multivariable linear regression, adjusted for each respective pre-intervention parameter, *p* < 0.05 considered as statistically significant.

BW, body weight; FOG, freezing of gait; MDS-UPDRS, Thai-translated versions of the Movement Disorder Society-Unified Parkinson Disease Rating Scale; SD, standard deviation; Kpa, Kilopascal; FTI, Force Time Integral.

Green highlights represent parameters, which are significantly affect from pre-intervention parameters.

Yellow highlight represents minor change of *p*-value from pre-intervention adjustment.

**Figure 2:**
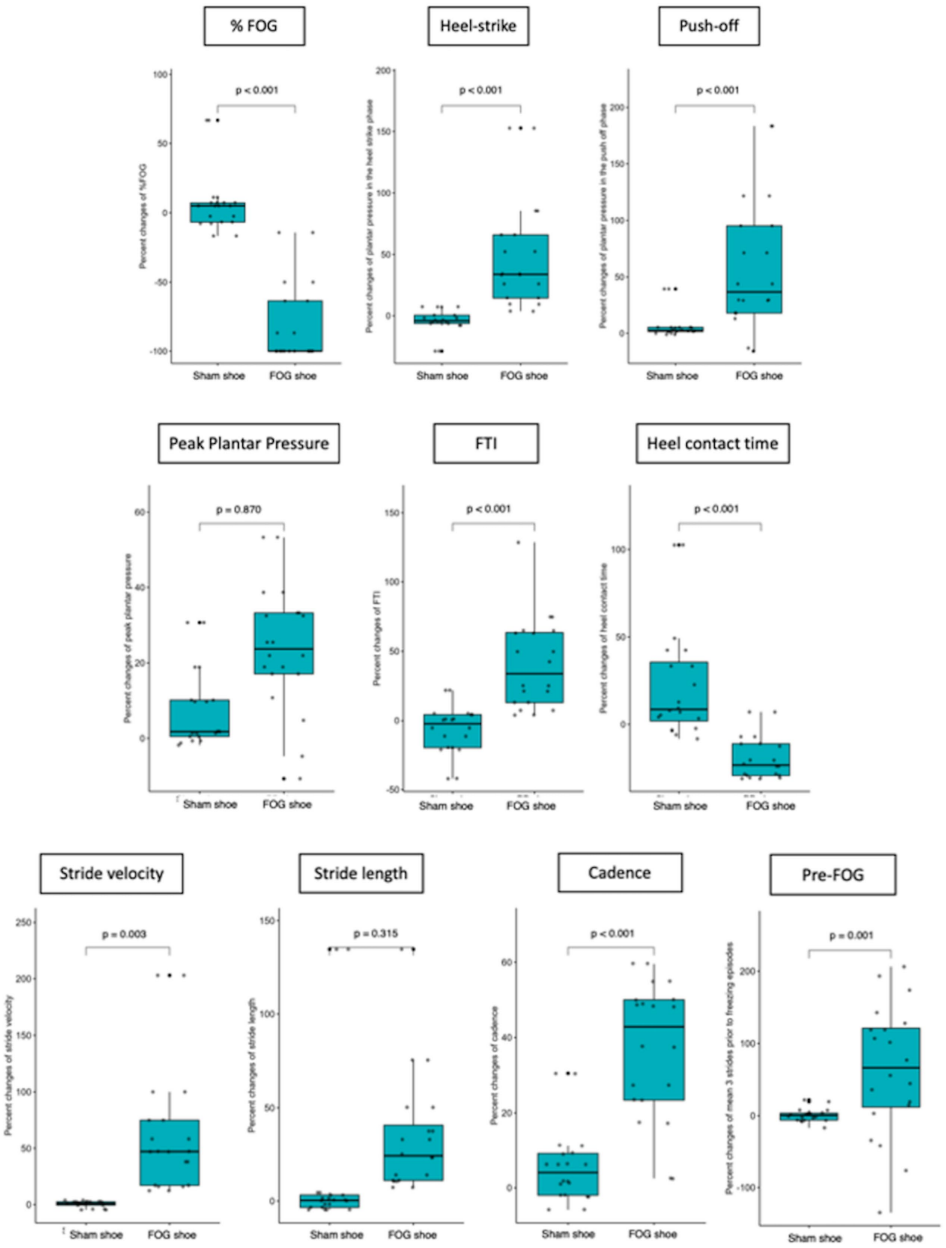
Comparison of FOG outcomes, plantar pressure, and spatiotemporal gait parameters before and after patients applied the shoes (both PD and sham shoes).

When focusing on the plantar pressure analysis, patients in the FOG shoes group exhibited significantly increased plantar pressure in the heel-strike (47.8 ± 43.7% vs. 4.9 ± 10.3%, *p* < 0.001, Cohen’s *d* = 1.35), plantar pressure in the push-off (56.3 ± 60.9% vs. 6.2 ± 11.6%, *p* < 0.001, Cohen’s *d* = 1.14), FTI (40.9 ± 32.5% vs. 6 ± 40.9%, *p* < 0.001, Cohen’s *d* = 0.94), and decreased heel contact time (19.3 ± 12.3% vs. 22.7 ± 32.5%, *p* < 0.001, Cohen’s *d* = 0.14) when compared with the sham group (Figure 2; Table 2). There was no significant difference of peak plantar pressure (27.2 ± 69.1% vs. 0.4 ± 29.5%, *p* = 0.870) between two groups. The change of peak plantar pressure after stimulation was variable in both groups. 8/20 or 40% in the sham group and 11/20 or 55% in the FOG shoe group showed decrease in peak plantar pressure (see Supplementary Figure 1).

Regarding spatiotemporal gait parameters, patients who applied FOG shoes demonstrated increased stride velocity (61.4 ± 55.3% vs. 0.5 ± 2.7%, *p* = 0.003, Cohen’s *d* = 1.56), cadence (37 ± 18.1% vs. 5.6 ± 10%, *p* < 0.001, Cohen’s *d* = 2.15), and stride length in the three strides before freezing episodes (62.7 ± 91.2% vs. 0.7 ± 9.1%, *p* = 0.001, Cohen’s *d* = 0.96). However, the normalized percent change of stride length, the CV of stride length and three strides before a freezing episode did not reach statistical significance among patients who were treated with FOG shoes and those treated with sham shoes (Figure 2; Table 2).

**Figure 3:**
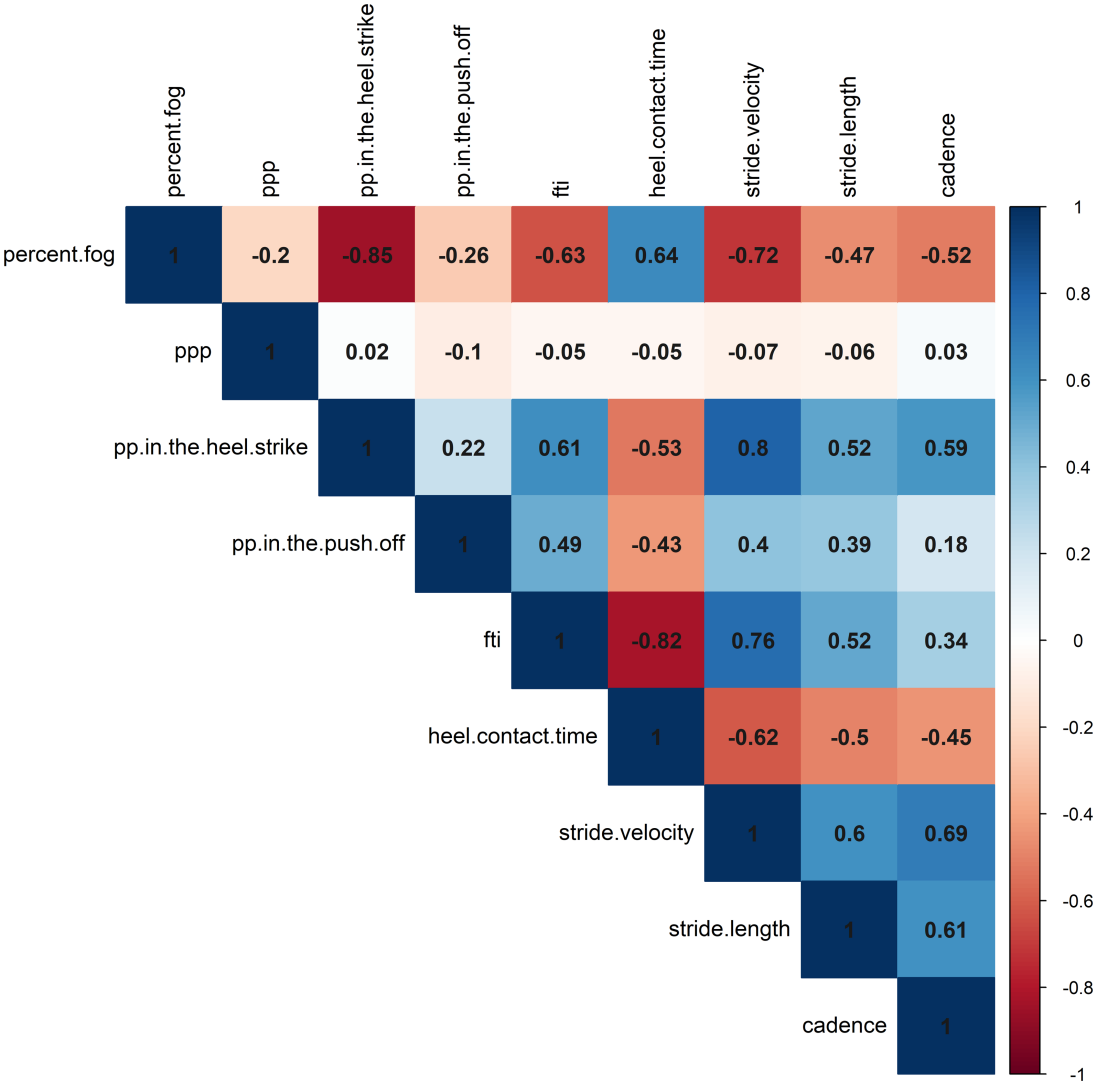
A correlation heatmap representing the correlations between objective FOG parameters, plantar pressure, and spatiotemporal gait parameters.

Exploratory correlation analyses were conducted between the normalized percent change of %FOG and plantar pressure analysis. Our study revealed negative correlations between %FOG, plantar pressure in the heel-strike (*r* = −0.847, *p* < 0.001) and FTI (r = −0.632, *p* < 0.001). A positive correlation was observed between %FOG and heel contact time (*r* = 0.638, *p* < 0.001). Furthermore, correlation analyses were also performed between normalized percent change of %FOG and spatiotemporal gait parameters. Our findings revealed negative correlations between %FOG and stride velocity (*r* = − 0.720, *p* < 0.001), stride length (*r* = − 0.469, *p* = 0.001) and cadence (*r* = − 0.518, *p* = 0.001). However, no significant correlation was found between %FOG and the CV of stride velocity and three strides before a freezing episode (*r* = −0.216, *p* = 0.181; r = 0.013, *p* = 0.938) (Figure 3; Table 3).

**Table 3:** Correlations between objective FOG parameters, plantar pressure, and spatiotemporal gait parameters.

Parameters	Δ Percent FOG (%)		Δ Peak plantar pressure (Kpa)		Δ Plantar pressure in the heel- strike (%BW)		Δ Plantar pressure in the push-off (%BW)		Δ FTI (%BW* Seconds)		Δ Heel contact time (seconds)		Δ Stride velocity (cm/s)		Δ Stride length (cm)		Δ Cadence (steps/min)		Δ Mean stride lengths of three strides before a freeze (cm)		Δ Coefficient of Variation (CV) of stride velocity (%)		Δ Coefficient of Variation (CV) of stride length (%)		Δ Coefficient of Variation (CV) of three strides before a freeze (%)	
	*r*	*p*-value	*r*	*p*-value	*r*	*p*-value	*r*	*p*-value	*r*	*p*-value	*r*	*p*-value	*r*	*p*-value	*r*	*p*-value	*r*	*p*-value	*r*	*p*-value	*r*	*p*-value	*r*	*p*-value	*r*	*p*-value
Δ Percent FOG (%)	–	–	–	–	–	–	–	–	–	–	–	–	–	–	–	–	–	–	–	–	–	–	–	–	–	–
Δ Peak plantar pressure (Kpa)	−0.202	0.211	–	–	–	–	–	–	–	–	–	–	–	–	–	–	–	–	–	–	–	–	–	–	–	–
Δ Plantar pressure in the heel- strike (%BW)	−0.847	<0.001	0.016	0.922	–	–	–	–	–	–	–	–	–	–	–	–	–	–	–	–	–	–	–	–	–	–
Δ Plantar pressure in the push-off (%BW)	−0.257	0.109	−0.096	0.557	0.223	0.166	–	–	–	–	–	–	–	–	–	–	–	–	–	–	–	–	–	–	–	–
Δ FTI (%BW*Seconds)	−0.632	<0.001	−0.049	0.766	0.611	<0.001	0.491	0.001	–	–	–	–	–	–	–	–	–	–	–	–	–	–	–	–	–	–
Δ Heel contact time (seconds)	0.638	<0.001	−0.049	0.765	−0.528	<0.001	−0.434	0.005	−0.821	<0.001	–	–	–	–	–	–	–	–	–	–	–	–	–	–	–	–
Δ Stride velocity (cm/s)	−0.720	<0.001	−0.070	0.667	0.802	<0.001	0.401	0.010	0.764	<0.001	−0.620	<0.001	–	–	–	–	–	–	–	–	–	–	–	–	–	–
Δ Stride length (cm)	−0.469	0.002	−0.061	0.709	0.521	<0.001	0.387	0.013	0.516	<0.001	−0.496	0.001	0.605	<0.001	–	–	–	–	–	–	–	–	–	–	–	–
Δ Cadence (steps/min)	−0.518	0.001	0.032	0.844	0.588	<0.001	0.180	0.266	0.338	0.033	−0.448	0.004	0.695	<0.001	0.605	<0.001	–	–	–	–	–	–	–	–	–	–
Δ Mean stride lengths of three strides before a freeze (cm)	0.013	0.938	0.029	0.856	−0.095	0.558	0.052	0.749	0.087	0.591	−0.067	0.681	−0.049	0.763	−0.230	0.152	−0.258	0.107	–	–	–	–	–	–	–	–
Δ Coefficient of Variation (CV) of stride velocity (%)	−0.216	0.181	0.040	0.806	0.241	0.134	−0.026	0.872	0.134	0.408	−0.077	0.633	0.125	0.443	0.161	0.319	0.007	0.965	−0.053	0.743	–	–	–	–	–	–
Δ Coefficient of Variation (CV) of stride length (%)	0.450	0.003	−0.231	0.152	−0.383	0.014	−0.215	0.183	−0.352	0.025	0.128	0.431	−0.532	<0.001	−0.287	0.072	−0.171	0.292	−0.004	0.976	−0.037	0.819	–	–	–	–
Δ Coefficient of Variation (CV) of three strides before a freeze (%)	0.340	0.031	−0.139	0.391	−0.268	0.095	−0.139	0.389	−0.295	0.064	0.063	0.698	−0.318	0.045	−0.217	0.178	−0.095	0.556	0.025	0.877	−0.078	0.630	0.457	0.003		–

Positive correlations were also observed between plantar pressure in the heel-strike and stride velocity (*r* = 0.802, *p* < 0.001), stride length (*r* = 0.521, *p* = 0.001) and cadence (*r* = 0.588, *p* < 0.001), and mean stride lengths at pre-FOG (*r* = 0.434, *p* = 0.005). Correlations between plantar pressure in the push-off and spatiotemporal gait parameters were observed only in relation to stride velocity (*r* = 0.401, *p* = 0.01) and stride length (*r* = 0.521, *p* < 0.001). Our study also demonstrated significant correlations between FTI and heel contact time with stride velocity, stride length, and cadence (Figure 3; Supplementary Table 1).

## 6 Discussion

Our study demonstrated the immediate effect of combination of static vibratory and plantar pressure stimulation integrated in the FOG shoe while seated in comparison to sham shoes for PD patients experiencing FOG. Consistent with our previous findings, there were significant improvement in %FOG, ameliorations in TUG scores, improvement of spatiotemporal gait parameters and the mean stride lengths of three strides prior to the freezing episode among individuals wearing static FOG shoes when compared to those applying sham shoes. Interestingly, our novel findings showed that patients employing FOG shoes displayed significantly increased plantar pressure the heel-strike and push-off of the gait cycle, FTI, and heel contact time in contrast to those with sham shoes, leading to the fact that manipulation of the peripheral nervous pathway could be able to alleviate FOG.

The immediate residual effect of FOG shoes incorporating static vibratory and plantar pressure stimulation was demonstrated in alleviating FOG among PD patients. Notably, significant improvements in %FOG, TUG scores and spatiotemporal gait parameters (stride velocity, cadence, mean stride lengths of three strides prior to the freezing episode) were observed when comparing FOG shoes to sham shoes. These improvements may be attributed to enhanced proprioceptive processing. The residual Aβ cutaneous mechanoreceptors could be stimulated by a soft silicone pressure pad with thickened silicone at the first hallux, the first metatarsal bone, and the sole, while muscle spindles at the Achilles tendon are stimulated by vibration (Ribot-Ciscar et al., 1998; De Nunzio et al., 2010). These combined signals from different receptors may ascend through the posterior column of the spinal cord and stimulate the sensorimotor cortices, thereby contributing to the restoration of gait (Pereira et al., 2016). Another possible mechanism is a cue-related effect. The integration of combined sensory stimulation within the FOG shoes may act as an external cue, compensating for the dysfunction of internal cues, and improving stride velocity and the mean three stride lengths during the pre-freezing episode (Iansek et al., 2006; Takakusaki, 2013). This cue-related effect may also stimulate sensorimotor cortical activation, contributing to the improvement of APA in PD patients, and resulting in resolution of FOG (Iansek et al., 2006). However, due to limitations in the current experimental design that the combined plantar stimulation was not synchronized to the gait cycle, the primary mechanism of the FOG shoe in improving FOG is the direct effect of combined peripheral plantar stimulation on muscle spindles rather than the cue-related effect. Interestingly, contrary to our previous study, stride length and gait variability did not exhibit significant changes in PD patients applying FOG shoes compared to those with sham shoes. This discrepancy could be attributed to the inclusion of patients with advanced disease stages and longer disease duration in the present cohort.

In terms of novel discoveries, our study has revealed significant improvements in plantar pressure during the heel-strike and push-off phases, accompanied by alterations in heel contact time and FTI among PD patients utilizing FOG shoes. These findings parallel earlier studies on plantar pressure analysis, which demonstrated reduced heel-strike, increased midfoot pressure, and delayed push-off in PD patients when compared to controls (Nieuwboer et al., 1999; Zou et al., 2023). The observed increase in heel-strike pressure resulting from the combined vibratory and pressure stimulation might originate from enhanced vibration-induced deceptive perception in the opposite direction. This could augment the contraction of the tibialis anterior muscle and ankle flexion during the initial gait phases, thereby influencing stride velocity and overall gait performance in PD patients (Courtine et al., 2007; De Nunzio et al., 2010). This enhanced ankle and knee flexion posture could contribute to forward body displacement and, consequently, an increase in stride velocity, indicative of improved proprioceptive processing and increased stimulation of parieto-cortical region, or even an enhanced APA (Carvalho et al., 2020). The increased peak plantar pressure during push-off, reduced heel contact time from accelerated stride velocity, and the positive correlations detected between plantar pressure during heel-strike and stride velocity, further support these hypotheses. Even the plantar pressure during heel-strike and push-off were increased in the active stimulation group but the overall peak plantar pressure did not show significantly different between groups. This observation could be attributed to the counterbalance between plantar pressure during push-off and heel-strike in the different phase of gait cycle, which might result from sensory gating (Cromwell et al., 2008; Cheng et al., 2016).

**Figure 4:**
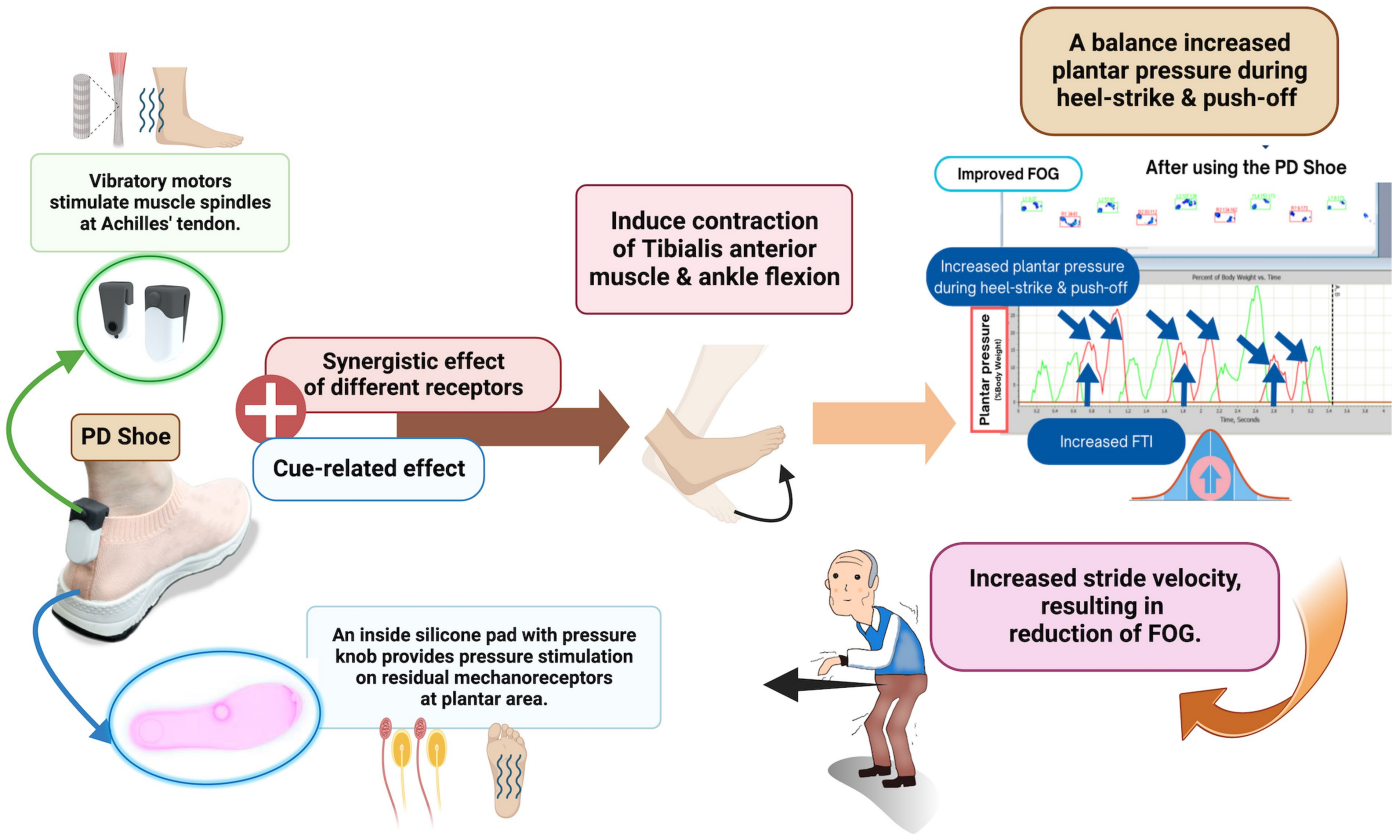
Proposed mechanisms of action of the FOG shoe. Created with BioRender.com.

While the enhanced knee flexion posture might initially raise concerns about worsening FOG, our study has demonstrated an increase in FTI without changing the peak plantar pressure in the FOG shoes arm compared to the sham shoes (Nieuwboer et al., 1999). This outcome indicates that the increased plantar pressure during push-off is counterbalanced by an increased plantar pressure during heel-strike. The nhanced APA resulting from combined sensory stimulations might also facilitate the correction of the flexion posture. Therefore, the potential deleterious effect of enhanced knee flexion posture is alleviated, resulting in an increased stride velocity, and reduction of FOG. Additionally, stride velocity was increase with while the stride length was not significantly changed with combined plantar stimulation group. This could imply that combined peripheral plantar stimulation may have a more pronounced positive effect on resetting the walking speed rather than the walking length. These observations are also supported by the negative correlations between FOG severity and plantar pressure during heel-strike. Notably, the correlations between FOG severity and plantar pressure during heel-strike are significantly stronger compared to those during push-off. Based on these results, we propose that combined peripheral plantar stimulation leads to an increase in plantar pressure during heel-strike, contributing to an enhanced gait speed, and amelioration of FOG severity (Figure 4) (Ginis et al., 2017; Carvalho et al., 2020).

Since we could demonstrate that combined vibratory and pressure stimulation embedded in the shoes had the positive effect on FOG resulting from enhanced proprioceptive processing in the peripheral and central loops and improved sensory gating, our FOG shoes might be considered a new treatment option for FOG. Our prototype FOG shoes offer a versatile solution with the potential to enhance the daily lives of patients with PD. The combined stimulation incorporated within the FOG shoe can be easily administered when the patient activates a switch. In addition, FOG shoes can be adaptable across a spectrum of daily activities, from walking to running. The affordability of these shoes is also noteworthy, as it consists of widely available materials, with an approximate cost of 1,000 Thai Baht (equivalent to 25 US dollars), making them an economical yet impactful option accessible to a broader patient demographic, regardless of financial circumstances.

### Limitations

Despite the benefits and effectiveness of FOG shoes, a few limitations remain. Firstly, the potential effects arising from differences in pre-stimulation parameters between the sham and FOG shoe groups should be considered, particularly in MDS-UPDRS III and peak plantar pressure. These effects might be a result of blinded randomization. Therefore, we accounted for this difference by performing the statistical test in a post-hoc analysis. Secondly, our reported plantar pressure and spatiotemporal gait outcomes were the averaged responses for each parameter. These do not account for individual responses from those who still exhibited FOG/FOG that worsened, or from the non-responders to the combined plantar stimulation. This could have effects on several spatiotemporal parameter changes. However, the individual responses could be further elaborated upon in future studies to determine the predictors of responders or non-responders. Thirdly, the FOG shoes lack sensors for synchronizing combined plantar stimulations with the gait cycle, and patients wore the FOG shoes and took the shoes off prior to the gait assessment. The real-time effect of FOG shoes on the freezing episodes and plantar pressure are still unknown. Nevertheless, we could demonstrate the immediate effects of FOG shoes on FOG, plantar pressure, and the clinical score (TUG). Our findings on alterations in peak plantar pressure from the midfoot to heel-strike and increase push-off are significant to highlight the importance of peripheral sensory system in contributing to FOG. Fourthly, the limited sample size, though a reliable method was used for calculating the sample size, and our achieved sample size is comparable to similar preceding studies (Bernard, 2000; Pinto et al., 2018). In addition, PD patients who experienced frequent FOG from the FOG-Q were intentionally recruited. Lastly, the exact mechanism of the combined plantar stimulations on the pre-FOG periods in patients who still experienced FOG compared to those who did not have FOG was still unclear. It seems that the mean three stride lengths before a freezing episode was higher in no residual FOG group (defined by 100% change of % FOG) but not statistically significant (not shown). Further study with larger number of patients for subgroup analysis should be considered.

Future research should explore the real-world effectiveness of FOG shoes through home-based trials. Incorporating FOG shoes into daily living may improve quality of life by enhancing mobility, reducing FOG limitations, and promoting an active lifestyle in PD patients with FOG. The development of a second version of FOG shoes, equipped with integrated sensors and predictive algorithms, is being planned to enhance deeper insights into peripheral stimulation and its impact on pathophysiology of FOG. With this method, the combined plantar stimulation will be synchronized with the gait cycle. This may better augment the gait performance and normalize plantar pressure to overcome FOG. This system could be seamlessly integrated with smartphone or smartwatch applications to provide patients with FOG alerts. Furthermore, deliberations are ongoing regarding enhancements to battery capacity to ensure efficient rechargeability. Additionally, there is contemplation of further customization, including the adaptation of stimulation frequencies tailored to individual patients, and an expansion of the versatility of FOG shoes to encompass a range of activities such as sports, leisure pursuits, and formal events.

In summary, our study demonstrated that the FOG shoe with combined vibratory and pressure stimulation may serve as a potential alternative or adjunctive therapy for FOG. This is achieved through its effects on enhanced plantar pressure during the heel-strike and push-off phases, increased stride length and increase mean three stride length prior to the FOG onset. Furthermore, the observed benefits of the FOG shoes on plantar pressure emphasize the significance of combined peripheral plantar stimulations in augmenting the peripheral and central sensory system, and improving the sensory gating, thus ameliorating FOG in PD patients.

## Data availability statement

The original contributions presented in the study are included in the article/Supplementary material, further inquiries can be directed to the corresponding author.

## Ethics statement

The studies involving humans were approved by Human Ethics Committee at Chulalongkorn University’s Faculty of Medicine (Institutional Review Board [IRB] No. 097/64). The studies were conducted in accordance with the local legislation and institutional requirements. The participants provided their written informed consent to participate in this study.

## Author contributions

WP: Conceptualization, Data curation, Formal analysis, Investigation, Methodology, Project administration, Writing – original draft. PP: Conceptualization, Data curation, Formal analysis, Methodology, Project administration, Supervision, Writing – review & editing. PV: Conceptualization, Writing – review & editing. DS: Conceptualization, Writing – review & editing. RB: Conceptualization, Funding acquisition, Methodology, Project administration, Resources, Supervision, Visualization, Writing – review & editing.
