# Supplementary material for: Residual effects of combined vibratory and plantar stimulation while seated influences plantar pressure and spatiotemporal gait measures in individuals with Parkinson’s disease exhibiting freezing of gait
Source: Front Aging Neurosci. 2024 Jan 9;15:1280324. doi: 10.3389/fnagi.2023.1280324 (PMC10803580; doi:10.3389/fnagi.2023.1280324)
Supplement: Supplementary file 1 [file Data_Sheet_1.DOCX]

**Supplementary Table 1** Comparison between pre-stimulation parameters in each group

| **Outcomes** | **Sham shoe**  **(n=20)** | **FOG shoe**  **(n=20)** | ***p*-value** |
| --- | --- | --- | --- |
| **MDS-UPDRS**  **part III in**  **the “On” period** | 23.9 ± 8.2 | 32.8 ± 9.2 | 0.02* |
| **Time-Up-and-Go (seconds)** | 21.9 ± 9.6 | 33.6 ±17.7 | 0.165 |
| **Percent FOG (%)** | 23.9 ± 8.2 | 33.6 ±17.7 | 0.149 |
| **Peak plantar pressure (Kpa)** | 219 ± 9.6 | 149.7 ± 24 | <0.001 |
| **Plantar pressure**  **in the heel strike**  **phase (%BW)** | 28.7 ± 11.5 | 32.5 ± 23.6 | 0.678 |
| **Plantar pressure**  **in the push-off**  **(%BW)** | 29.6 ± 11 | 27 ± 23.7 | 0.265 |
| **FTI (%BW*Seconds)** | 24.1 ± 9.4 | 14.9 ± 13.2 | 0.014* |
| **Heel contact time (seconds)** | 0.4 ± 0.2 | 0.4 ± 0.1 | 0.121 |
| **Stride velocity (cm/s)** | 67.9 ± 15.5 | 44.8 ± 23.3 | 0.001* |
| **Stride length (cm)** | 70.6 ± 20.4 | 54.8 ± 27.8 | 0.114 |
| **Cadence (steps/min)** | 96.3 ± 17.9 | 77.5 ±12.1 | <0.001* |
| **Mean stride lengths of three strides before a freeze (cm)** | 48.3 ± 20.4 | 37.9 ±11.4 | 0.046* |
| **Coefficient of Variation**  **(CV) of stride velocity (%)** | 33.4 ± 4.7 | 42.8 ±17.7 | 0.341 |
| **Coefficient of Variation**  **(CV) of stride length (%)** | 9.3 ± 8.1 | 18.5 ± 18.9 | 0.004* |
| **Coefficient of Variation**  **(CV) of three strides  before a freeze (%)** | 4.8 ± 1.9 | 21.3 ± 25.2 | <0.001* |
| **p*-values from Unpaired t-test or Mann-Whitney U test, *p* < 0.05 considered as statistically significant  BW, body weight; FOG, freezing of gait; MDS-UPDRS, Thai-translated versions of the Movement Disorder Society-Unified Parkinson Disease Rating Scale; SD, standard deviation; Kpa, Kilopascal; FTI, Force Time Integral. | | | |
