# Supplementary figures and images for: Residual effects of combined vibratory and plantar stimulation while seated influences plantar pressure and spatiotemporal gait measures in individuals with Parkinson’s disease exhibiting freezing of gait
Source: Front Aging Neurosci. 2024 Jan 9;15:1280324. doi: 10.3389/fnagi.2023.1280324 (PMC10803580; doi:10.3389/fnagi.2023.1280324)

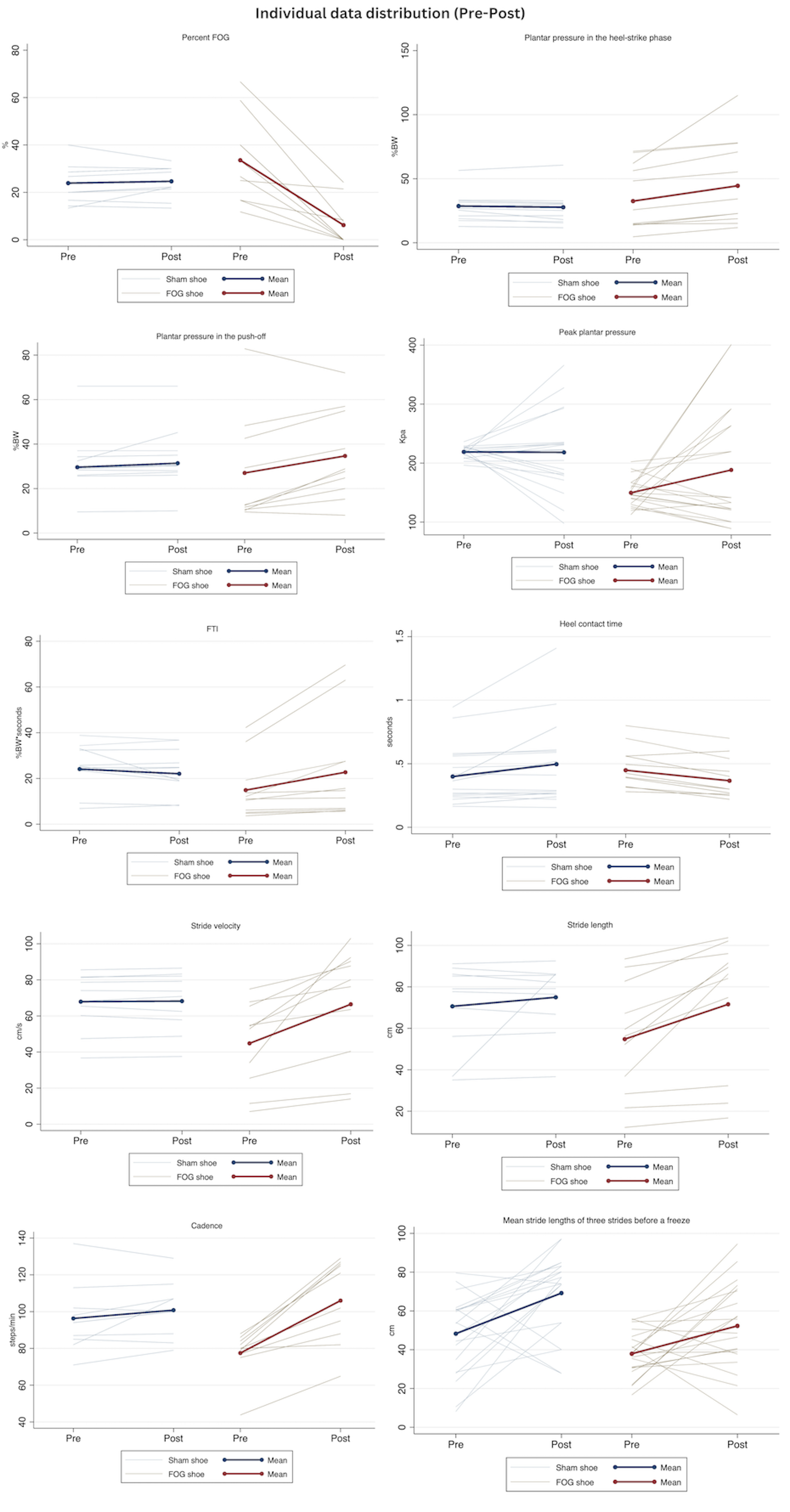

Supplement: Supplementary file 2 [file Image_1.TIFF]
